# A modified formulation of Huanglian-Jie-Du-Tang reduces memory impairments and β-amyloid plaques in a triple transgenic mouse model of Alzheimer’s disease

**DOI:** 10.1038/s41598-017-06217-9

**Published:** 2017-07-24

**Authors:** Siva Sundara Kumar Durairajan, Ashok Iyaswamy, Sravan Gopalakrishna Shetty, Ananth Kumar Kammella, Sandeep Malampati, Wenbin Shang, Chuanbin Yang, Juxian Song, Sookja Chung, Jiandong Huang, Kaliappan Ilango, Quan-Bin Han, Min Li

**Affiliations:** 1 Neuroscience Research Laboratory, Mr. & Mrs. Ko Chi-Ming Centre for Parkinson’s Disease Research, School of Chinese Medicine, Hong Kong Baptist University, Kowloon Tong, Hong Kong; 2 Mr. & Mrs. Ko Chi-Ming Centre for Parkinson’s Disease Research, School of Chinese Medicine, Hong Kong Baptist University, Kowloon Tong, Hong Kong; 3 Department of Anatomy, Li Ka Shing Faculty of Medicine, The University of Hong Kong, Pokfulam Road, Hong Kong; 4 Department of Biochemistry, Li Ka Shing Faculty of Medicine, The University of Hong Kong, Pokfulam Road, Hong Kong; 5 Phytochemistry and Analysis laboratory, Interdisciplinary Institute of Indian System of Medicine, SRM University, Kattankulathur, Kancheepuram India; 6 Natural Products Chemistry & Analysis Laboratory, School of Chinese Medicine, Hong Kong Baptist University, Kowloon Tong, Hong Kong

## Abstract

Alzheimer’s disease (AD) is a degenerative disorder typified by progressive deterioration of memory and the appearance of β-amyloid peptide (Aβ)-rich senile plaques. Recently we have identified a novel function of a patented formulation of modified Huanglian-Jie-Tu-Tang (HLJDT-M), a Chinese herbal medicine, in treating AD in *in vitro* studies (US patent No. 9,375,457). HLJDT-M is a formulation composed of Rhizoma Coptitis, Cortex Phellodendri and Fructus Gardeniae without Radix Scutellariae. Here, we assessed the efficacy of HLJDT-M on a triple transgenic mouse model of AD (3XTg-AD). Oral administration of HLJDT-M ameliorated the cognitive dysfunction of 3XTg-AD mice and lessened the plaque burden. In addition, biochemical assays revealed a significant decrease in levels of detergent-soluble and acid-soluble Aβ via decreasing the levels of full length amyloid-β precursor protein (FL-APP) and C-terminal fragments of APP (CTFs) in brain lysates of HLJDT-M-treated mice. HLJDT-M treatment also significantly reduced the levels of FL-APP and CTFs in N2a/SweAPP cells. In contrast, treatment using the classical formula HLJDT did not reduce the memory impairment of 3XTg-AD mice and, rather, increased the Aβ/Fl-APP/CTFs in both animal and cell culture studies. Altogether, our study indicates that HLJDT-M is a promising herbal formulation to prevent and/or cure AD.

## Introduction

Alzheimer’s disease (AD) is a serious and chronic gradual neurodegenerative disease which results in the deterioration of normal mental function affecting every aspect of life^1, 2^. AD is the most common cause of dementia, presently affecting about 50 million people worldwide^3^, with an associated annual cost of an astounding USD 810 billion in 2010^3^. Both numbers are projected to rise exponentially as the global population ages, potentially precipitating health, social and economic crises. Currently approved drugs for AD treatment provide only modest improvements and temporary symptomatic relief; an effective disease-modifying treatment for this devastating disease remains to be discovered^4^.

The key histopathological hallmarks of AD comprise the appearance of senile plaques and neurofibrillary tangles (NFTs), loss of synapses, brain inflammation, neuronal loss and eventual brain shrinkage^1, 2^. This pathogenesis suggests a multifactorial causation. If true, then an effective therapeutic approach against AD might be a set of concerted pharmacological treatments that concurrently target different AD pathologies including amyloid-β (Aβ) plaque deposits, abnormal inflammation and neuronal loss^5^. It is widely acknowledged that Aβ plays a key role in the pathogenesis of AD since the presence of Aβ plaques is the defining feature for the diagnosis of AD. The significance of Aβ in the etiology of AD has been validated in many *in vivo* and *in vitro* systems. Aβ peptides containing 40 (Aβ1-40) or 42 (Aβ1-42) amino acids are cleaved from full length amyloid precursor protein (FL-APP) by two proteases, β- and γ-secretases, which have been implicated in the cause of AD^1^.

Several traditional Chinese medicine (TCM) formulas have been well-documented in the Chinese literature as medicines for dementia. Based on the principles of TCM, these formulas address not only modifying the disease symptoms but also restoring and sustaining the body’s homeostasis^6, 7^.

Huang-Lian-Jie-Du-Tang (HLJDT) is a classical TCM recipe for treating inflammatory, cerebral and liver diseases^8^. Although there are few reports on the neuroprotective activity of HLJDT, the precise mechanism by which HLJDT affect learning and memory is not known. HLJDT is a preparation of Rhizoma coptidis (RC), Radix scutellariae (RS) Cortex phellodendri (CP) and Fructus gardeniae (FG), in a 3:2:2:3 dry weight ratio^9^. Recently we found that berberine, a pure compound isolated from RC and CP, greatly attenuates the Aβ load in a transgenic AD mice by regulating APP processing^10^. In contrast, we have recently shown that baicalein, a pure compound in RS, increased the Aβ load in AD mice^11^. In that same study, we reported that the classic formula of HLJDT containing RS enhances the amyloid β-peptide (Aβ) generation in N2a cells stably expressing Swedish APP (N2a-SwedAPP) cells; this means that RS reduces the ability of HLJDT to reduce the generation of Aβ^8^. However, a modified HLJDT (HLJDT-M), a novel patented formulation prepared from RC, CP and FG without RS, significantly reduced the generation of amyloid-β (Aβ) by modulating amyloid precursor protein (APP) processing in N2a-Swed APP cells^11, 12^. Since these findings are from an *in vitro* AD model, we cannot be certain that HLJDT-M will have a similar Aβ-decreasing effect *in vivo*. Therefore, the aims of the present study are to test whether the treatment of HLJDT-M substitutes the Aβ increasing effects of HLJDT in 3XTg-AD transgenic mouse model, and on the regulatory processing of APP, thereby demonstrating a significantly more potent treatment for neurodegenerative diseases that does not associate with any Aβ increasing effect.

## Results

**Figure 1:**
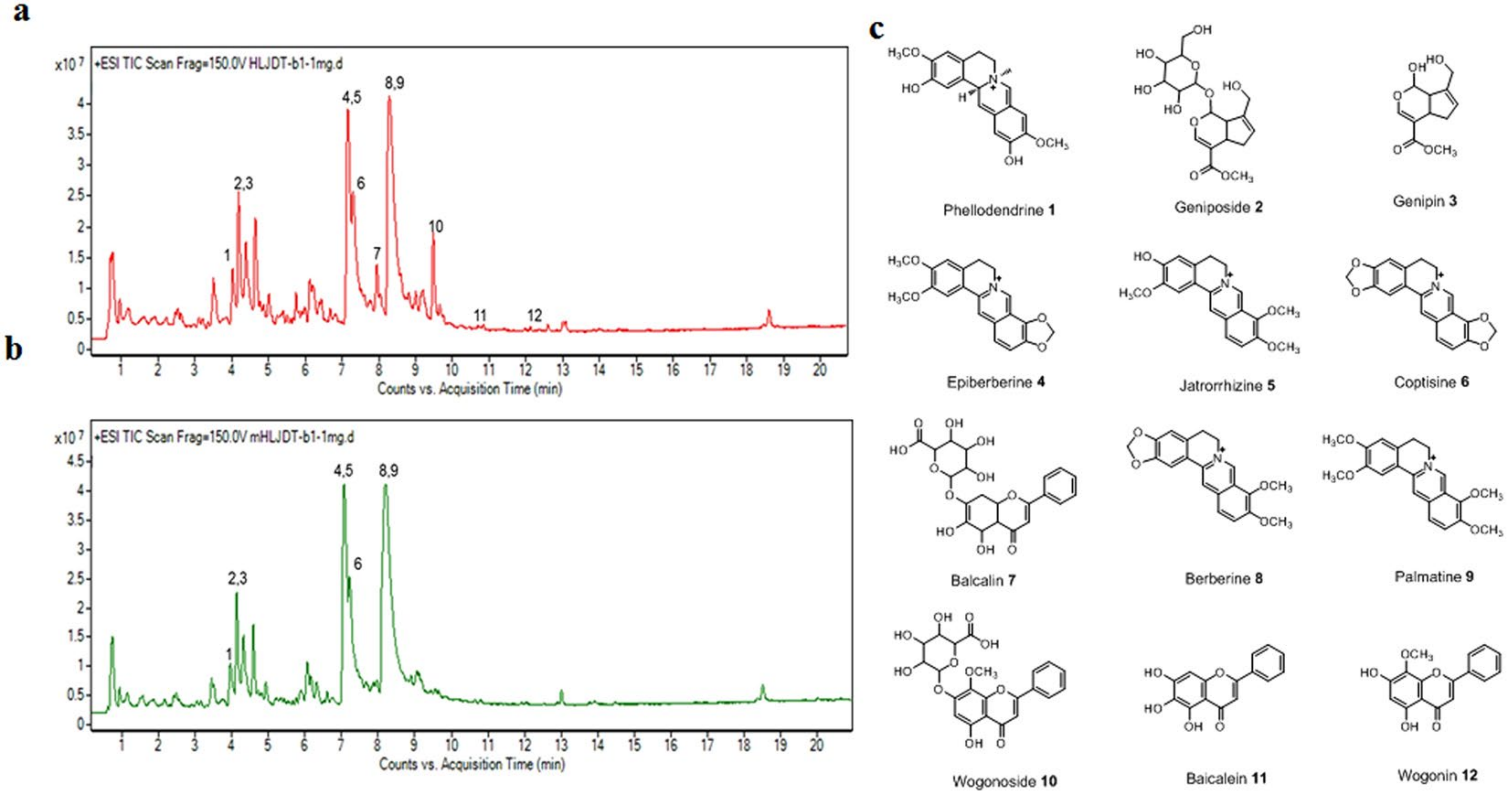
LC-ESI-Q/TOF chromatograms (TIC) of HLJDT (**a**) and HLJDT-M (**b**). (**c**). Chemical structure of representative compounds of HLJDT. Peaks: 1. Phellodendrine, 2. Geniposide, 3. Genipin, 4. Epiberberine, 5. Jaterorhizine, 6. Coptisine, 7. Baicalin, 8. Berberine, 9. Palmatine, 10. Wogonoside, 11. Baicalein, 12. Wogonin.

We first analyzed HLJDT and HLJDT-M by LC-qTOF/MS. The total ion current (TIC) chromatograms of HLJDT and HLJDT-M and their corresponding to positive ions are shown in Fig. 1 and Supplementary Table 1, respectively. The base peak chromatograms of the chemical fingerprints from the three batches of HLJDT and HLJDT-M were quite similar, demonstrating that HLJDT and HLJDT-M were produced regularly with good quality (data not shown). Twelve of the most abundant 24 peaks were identified and confirmed by comparison with external chemical markers, high-resolution MS and MS/MS fragmentation. The rich extraction efficiency and the three batches produced similar profiles of the phytochemical profiles from the three batches of samples suggested that these 12 compounds were a relevant representation of the phytochemical components of HLJDT and suitable for use as references.

### HLJDT-M or HLJDT does not affect weight and locomotor activity of 3XTg-AD mice

The chronic oral administration of HLJDT or HLJDT-M in 3XTgAD mice at doses up to 2 g/kg per day for 6 months neither reduce the body weight nor cause any notable harmful side effects (Supplementary Fig. 1a,b,c), so we concluded that both preparations were well tolerated. We then used the Open Field test to assess and compare the exploratory behavior and locomotor activity of untreated 3XTg-AD mice and those treated with HLJDT and HLJDT-M. No significant differences were observed between the vehicle and HLJDT- or HLJDT-M-treated mice in total moving distance, total ambulatory movement duration and velocity (Supplementary Fig. 2a,b,c). These results indicate that neither HLJDT nor HLJDT-M affected locomotor activity and exploratory behaviors.

### HLJDT-M but not HLJDT ameliorates deficits in spatial learning and memory in 3XTg-AD mice

**Figure 2:**
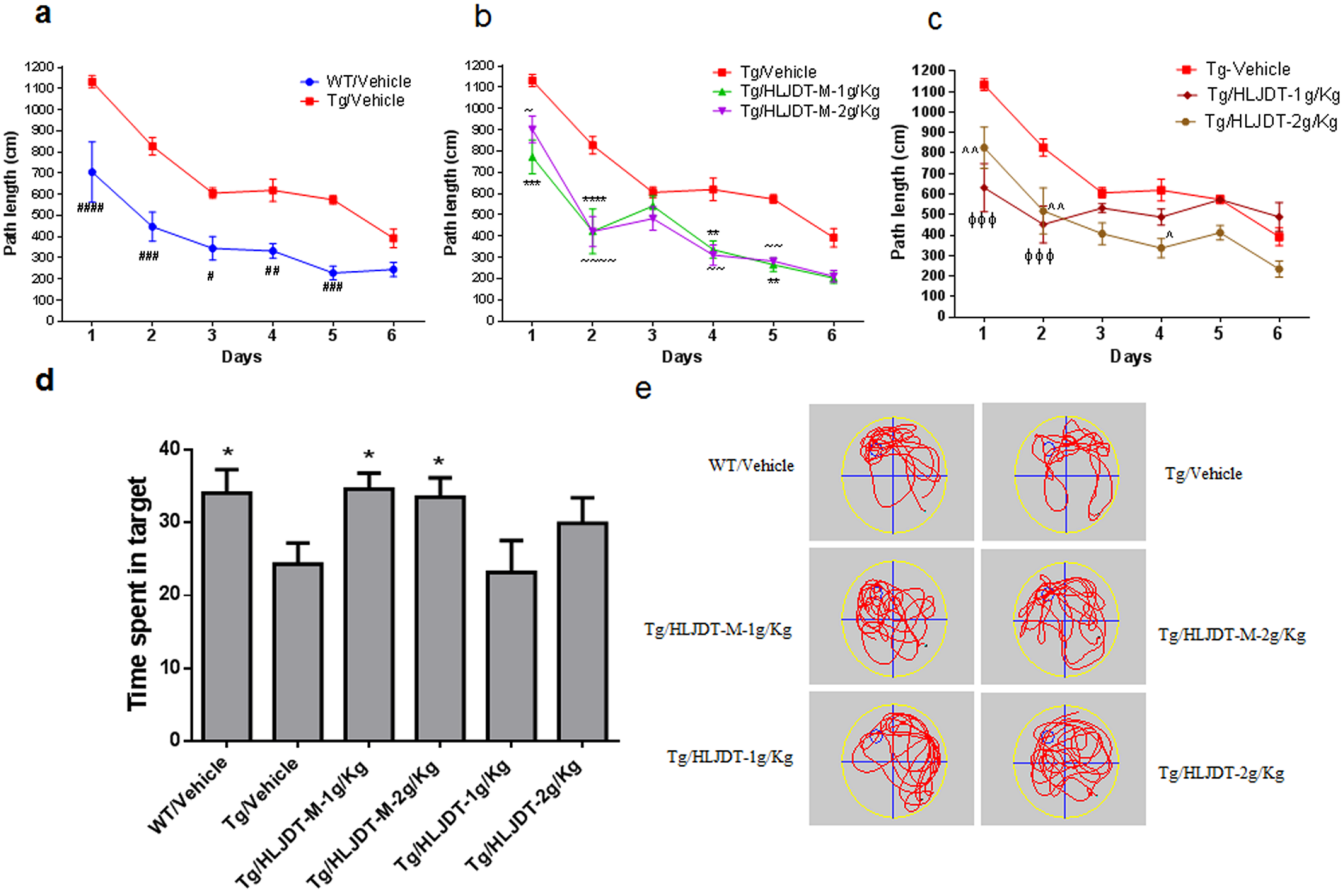
Behaviour study of HLJDT and HLJDT-M on 3XTg-AD mice. Acquisition of spatial memory was evaluated by Morris water maze (hidden platform) in HLJDT, HLJDT-M and vehicle-treated 3XTg-AD mice (**a**,**b**,**c**). Results are represented as the mean length values ± standard error of the mean (SEM) of all mice from 4 trials per day (n = 10). In probe trial, mice are evaluated for the amount of time spent in searching the platform location after 24-hour retention trial in MWM (**d**,**e**). In the probe trial, the HLJDT-M treated mice stayed longer in the target quadrant than the vehicle-treated mice, showing memory retention. The symbol denotes statistical differences among the given groups over all trial days. ^###^p < 0.001 (WT treated with vehicle vs. Tg treated with vehicle); ***p < 0.001 (Tg treated with HLJDT-M at 1 g/kg vs. Tg treated with vehicle), ~~~p < 0.001 (Tg treated with 2 g/kg of HLJDT-M vs. Tg treated with vehicle), ^^p < 0.01 (Tg treated with 1 g/kg of HLJDT vs. Tg treated with vehicle) and ^ΦΦΦ^p < 0.001 (Tg treated with 2 g/kg of HLJDT vs. Tg treated with vehicle).

As revealed by the Morris Water Maze (MWM) test, the chronic administration of HLJDT-M for 6 months in 3XTg-AD mice significantly ameliorated memory and spatial learning deficits of 3XTg-AD mice (Fig. 2b). The Supplementary Figure 3 shows that the WT, HLJDT-M, HLJDT and Tg control groups had a comparable path length in all trial blocks of the visible platform task [F(3,24) = 0.806; p = 0.51] suggesting that HLJDT-M or HLJDT treatment did not significantly influence motor function, visual acuity or motivation in all treated mice. On the next day, the platform was hidden and its position maintained until the end of the task.

Since Parachikova *et al*. has already shown that the 3XTg-AD mice normally take a greater distance to reach the platform than the age-matched WT mice in the MWM test^13^, we confirm that our 3XTg-AD animal colony also took a longer path to reach the platform than did the vehicle-treated WT-type mice (Fig. 2a). Vehicle-treated 3XTg-AD mice showed a lengthier path distance [F(5,36) = 27.8; p < 0.001] as compared to the WT mice over a 6-day training period (Fig. 2a). A genotype effect [F(1,36) = 86.2; p < 0.001] indicates a difference in path distance in the vehicle-treated 3XTg-AD groups compared with wild type (WT) animals from the 1^st^ to 5^th^ day trials (post-hoc, p < 0.05) (Fig. 2a).

In contrast, treatment of the 3XTg-AD mice with HLJDT-M noticeably decreased their path length when compared to vehicle-treated 3XTg mice as evidenced by the shorter path length these mice took on the 4^th^ and 5^th^ day learning test trials (Fig. 2b). Two-way ANOVA showed a significant interaction of treatment and days [F(5,72) = 58.3; p < 0.001] on the learning test days to a level similar to that of WT mice (Fig. 2a). Repeated two-way ANOVA also showed a statistical effect of treatment (1 g/kg and 2 g/kg doses of HLJDT-M) on the path distance of 3XTg-AD mice [F(3,72) = 36.6; p < 0.001]. Using Bonferroni’s post hoc test for multiple comparisons, during the trials from day 1 to day 6, HLJDT-M-treated 3XTg-AD mice learned significantly better than vehicle-treated 3XTg-AD mice at trial days 1, 2, 4 and 5, indicating that spatial learning was impaired in vehicle-treated, but not impaired in HLJDT-M-treated, 3XTg-AD mice. However, long-term treatment with HLJDT at 1 g/kg or 2 g/kg did not reverse impairments in spatial memory in 3XTg-AD mice (Fig. 2b), as shown at trial days 3–6. In post hoc multiple comparisons, from trial days 3–6, HLJDT-treated 3XTg-AD mice did not perform statistically differently from vehicle-treated 3XTg-AD mice (p > 0.05), indicating that spatial learning was not ameliorated in HLJDT-treated animals, but it was ameliorated in HLJDT-M-treated 3XTg-AD mice.

**Figure 3:**
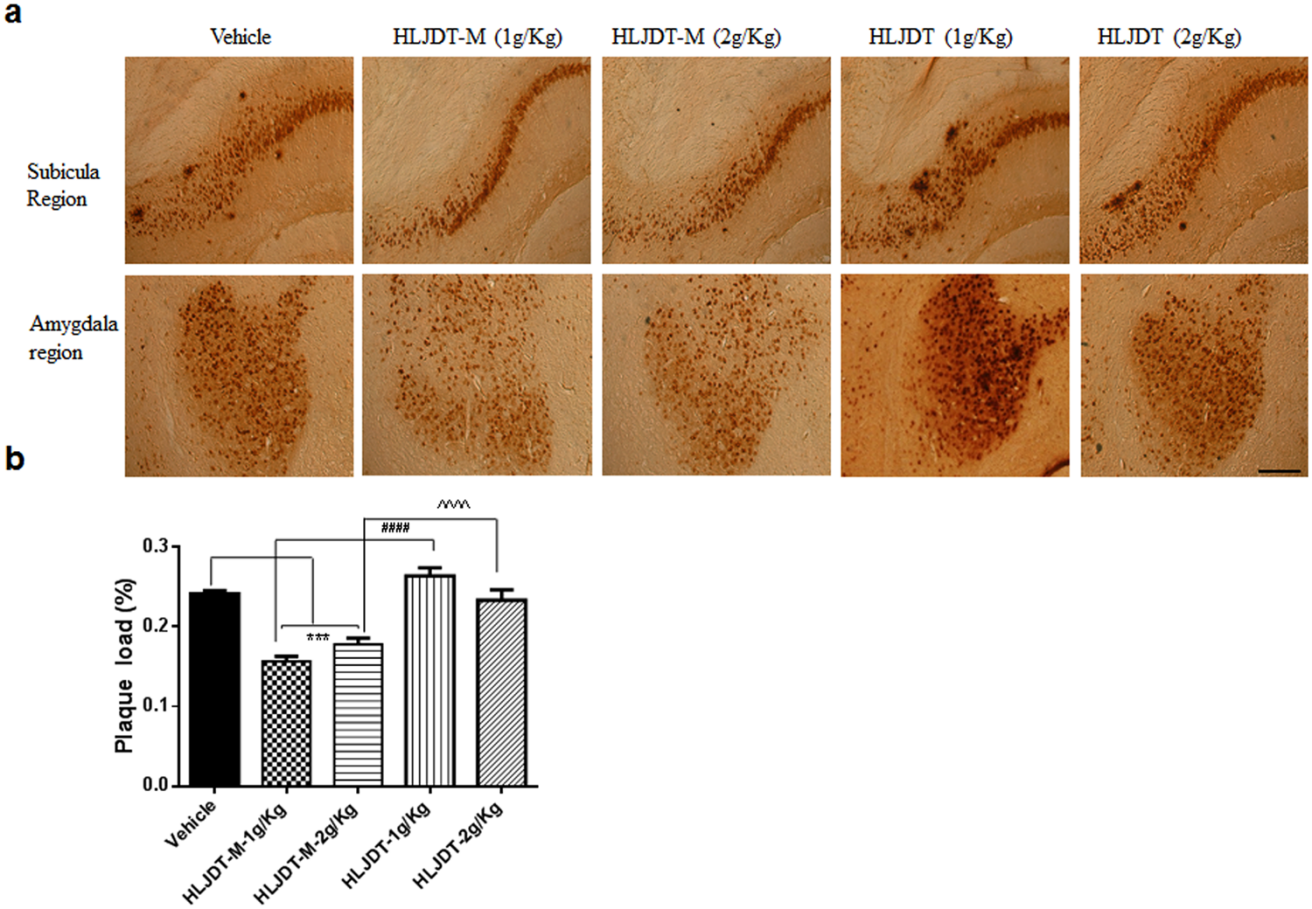
HLJDT-M, but not HLJDT, reduces hippocampal Aβ-plaque pathology in 3XTg-AD mice. Immunohitochemical labelling of Aβ with 4G8 antibody in representative figures taken from the coronal sections in 3XTg-AD mice were orally administered with HLJDT-M or HLJDT at doses of 1 and 2 g/kg per day for 6 months.

In order to measure memory retention of spatial learning, we did a probe trial 24 h after the 6^th^ hidden day. On the probe trial day, the HLJDT-M treated 3XTg-AD mice took longer amount of time probing for the platform in the target quadrant than did the vehicle and HLJDT-treated groups (Fig. 2c,d). One-way ANOVA analysis of the time spent in the target quadrant indicated a significant interaction of HLJDT-M treatment groups and probe trial [F(5,54) = 2.50; p < 0.05], suggesting that the HLJDT-M-treated 3XTgAD mice significantly increased their spatial bias in the target quadrant. The multiple comparisons by Fisher’s LSD test showed that the HLJDT-M treatment group (p < 0.05) but not the HLJDT treatment group (p > 0.05) differed significantly from the vehicle treatment group. Vehicle-treated WT mice also spent longer amount of time in the target quadrant (p < 0.05) than vehicle-treated 3XTg-AD mice. (Fig. 3c,d). Since the HLJDT-M-treated mice exhibited more localized search patterns in the target quadrant than vehicle and HLJDT-treatment groups over the location of hidden platform, we conclude that both spatial memory and memory retention improved in HLJDT-M-treated mice^14^. Altogether, the above findings revealed that spatial reference memory and long-term memory retention impairment of 3XTg-AD mice is ameliorated only by chronic treatment with HLDT-M but not with HLJDT.

### Chronic HLJDT-M but not HLJDT administration decreases plaque burden in 3XTg-AD mice

Because different batches of our colony of 3XTg-AD mouse show difference in Aβ pathology onset, we tested the development of Aβ plaques using mice at 6, 9, 12, 16, 18, and 24 months of age (n = 5 at each age) and found similar progressions of pathology in our mouse colony (data not shown) compared with the founder 3XTg-AD mouse colony^15^. At the commencement of treatment, i.e., 7 months of age, only the intracellular Aβ plaque was mostly prevalent in the subiculum and cerebral cortical region. The levels of intraneuronal Aβ-bearing neurons and the appearance of extracellular Aβ plaques were augmented with aging, and more advanced plaques was observed at 12-months age, i.e. near the endpoint of this study. No extracellular or intracellular Aβ immunostaining was observed in brain sections from non-transgenic mice (data not shown).

Although the HLJDT-M treatment decreased memory impairment in 3XTg-AD mice, intraneuronal Aβ and plaques in the brain are also relevant to neurodegeneration in 3XTg-AD mice^16^. Therefore, to verify whether the decreased memory impairment by HLJDT-M observed in 3XTgAD mice is correlated with decreased plaque load, we performed a systematic, unbiased, stereological, immunohistochemical analysis of Aβ in 3XTg-AD brain sections. We stained nine sections per mouse brain, cut at 120 μm intervals, and evaluated the total area and area occupied by Aβ staining in each section. Overall plaque load was estimated as the ratio of the area occupied by plaques to the area inside a region of interest.

The plaque burden was decreased by about 35% (p < 0.0001) in mice treated with HLJDT-M (1 g/kg/d) compared to vehicle-treated mice, whereas treatment with 2 g/kg/d of HLJDT-M reduced the Aβ plaque load to 26% of vehicle-treated levels (p < 0.001) (Fig. 3a,b). However, the plaque load was increased by 109% in mice treated with HLJDT (1 g/kg/d) compared to vehicle-treated mice (Fig. 3a,b), whereas treatment with 2 g/kg/d of HLJDT did not influence the Aβ load. Representative Aβ-stained histology sections of vehicle- and HLJDT-M- and HLJDT-treated mice are shown in Fig. 3a and b at comparable brain regions. To sum up, these results demonstrate that, in this AD mouse model, HLJDT-M alleviates the amyloid plaque load while HLJDT does not.

### Differential effects of HLJDT-M and HLJDT on the level of Aβ peptides in 3XTgAD mice

**Figure 4:**
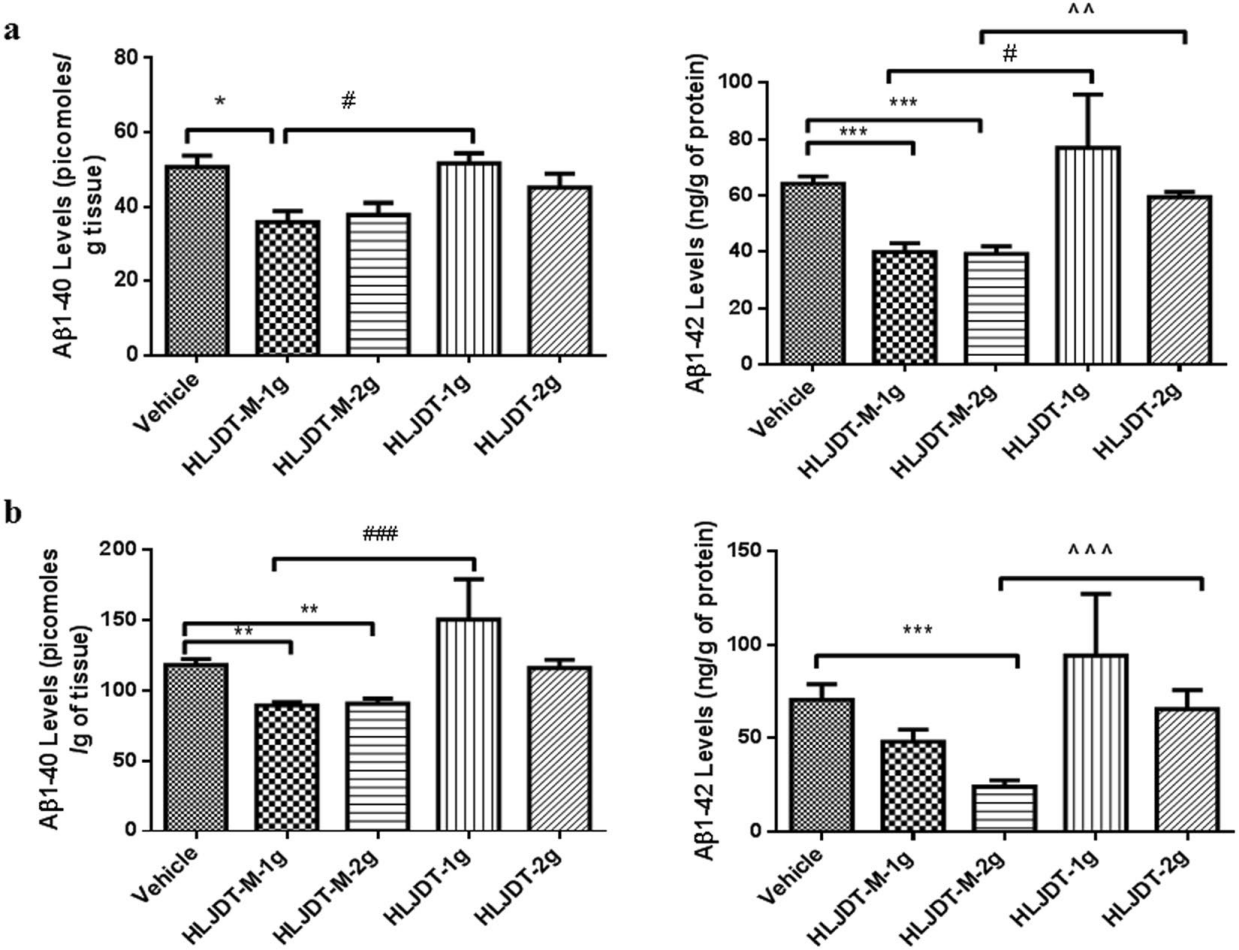
HLJDT-M but not HLJDT treatment reduces Aβ peptide levels in 3XTg-AD mice. SDS-soluble (**a**) and formic acid-soluble (**b**) Aβ1-40 and Aβ1-42 levels from the right brain hemisphere were measured by sandwich ELISA. Both Aβ1-40 and Aβ1-42 were reduced in the brains lysates of HLJDT-M-treated (**p < 0.01) animals. Compared to the HLJDT-M treatment groups, HLJDT significantly increased both detergent and acid-soluble Aβ1-40 and Aβ1-42 levels. Values denote group mean ± SEM. The statistical significant difference between the groups are denoted as *p < 0.05, **p < 0.01, ***p < 0.001 vs. vehicle control; ^#^p < 0.05, ^###^p < 0.001 for 1 g/kg HLJDT-M vs. 1 g/kg HLJDT and ^p < 0.05, ^^^p < 0.001 for 1 g/kg HLJDT-M vs. 1 g/kg HLJDT. N = 10 mice per group.

To further confirm the HLJDT-M-mediated reduction of 4G8-positive Aβ plaques in 3XTg-AD mice, the brain lysates were subjected to Aβ ELISA analysis. Detergent-soluble Aβ was initially extracted with 2% SDS by ultracentrifugation, and the resultant detergent-insoluble Aβ was then extracted with 70% formic acid after ultracentrifugation as previously described by Kawarabayashi *et al*.^17^ and by us^10^. Following 6 months of treatment, the amount of soluble and insoluble Aβ species in brain homogenates of 3XTg-AD mice were measured by ELISA and estimated in pmoles of Aβ per g of wet tissue weight. The level of Aβ species in the brain homogenates of WT mice B6129F2 cannot be calculated because the optical measurement is extremely low and much below the standard curve (data not shown). HLJDT-M treatment groups showed significant reduction [by 29% (p < 0.001)] in levels of soluble Aβ1-42 peptides when compared with vehicle-treated animals (Fig. 4a). Remarkably, these effects are connected with a great decrease in the levels of insoluble Aβ1-42 [by 34% (p < 0.001)], especially in the HLJDT-M group dosed with 2 g/kg per day (Fig. 4b). The HLJDT-M treatment slightly reduced (7%) the Aβ1-40 in both fractions; this reduction may represent an effect of HLJDT-M treatment on the specific clearance of Aβ1-42 (Fig. 4a,b).

In the HLJDT treatment groups, low-dose and high-dose HLJDT-treated animals had significantly increased levels of insoluble Aβ1-42 (195 and 271%, respectively), compared with the low-dose and high-dose of modified HLJDT-treated counterparts; however, high-dose HLJDT treatment did not significantly influence the levels of both compared with vehicle (Fig. 4a,b).

### HLJDT-M but not HLJDT reduces APP, CTFs, and their phosphorylation forms in the brains of 3XTg-AD mice

**Figure 5:**
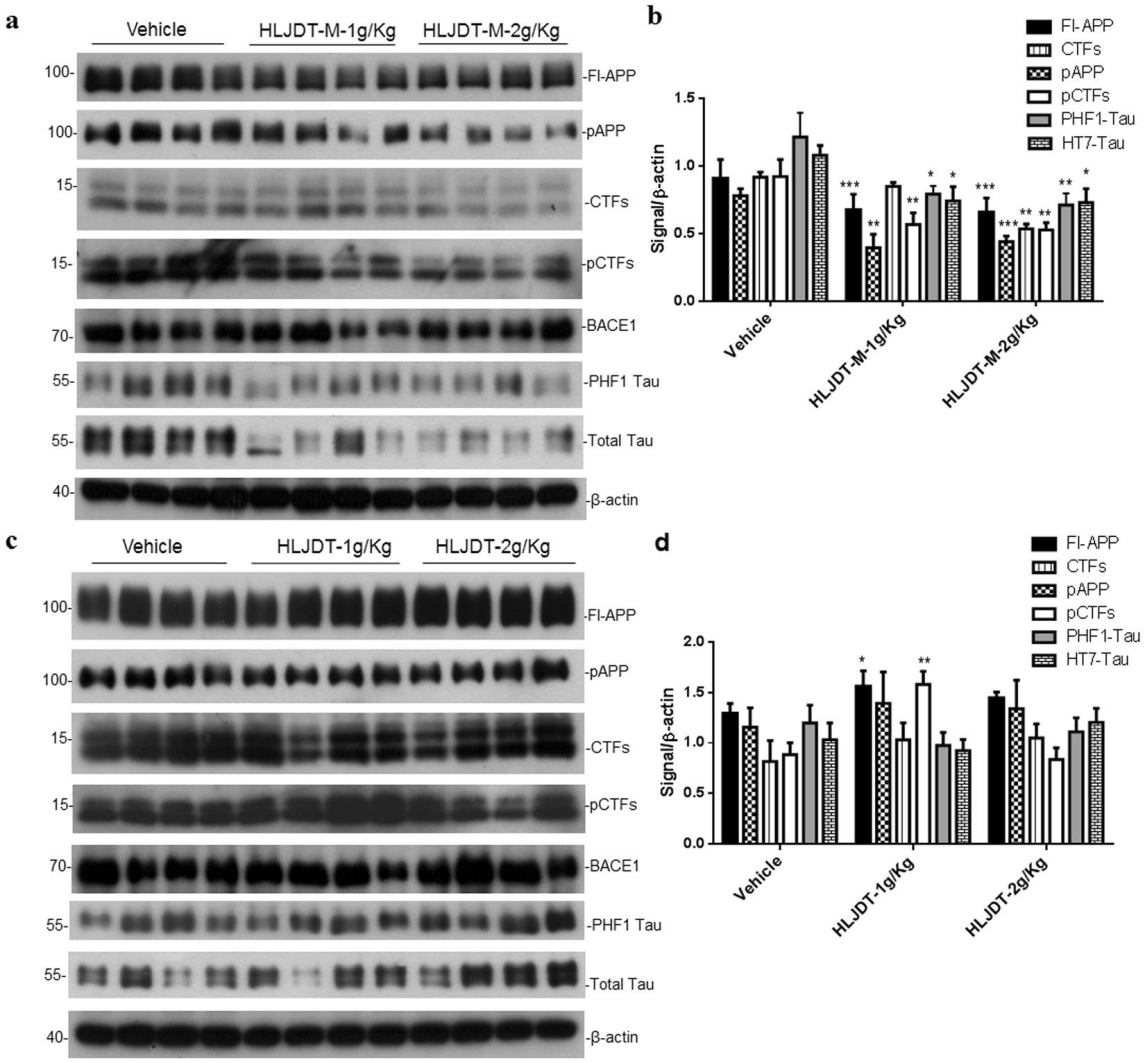
Different treatment effects of HLJDT-M (**a**) and HLJDT (**a**) on the levels of APP metabolites, phospho-Tau and total Tau in 3XTg-AD mice. Immunoblot presenting the levels of FL-APP, CTFs (CTFα and CTFβ), pAPP and pCTFs (α and β). Quantification of immunoblots by densitometric analysis and is presented as the ratio of FL-APP, CTFs, pAPP, PHF-1 Tau, total Tau and pCTFs against β-actin in the SDS brain lysates of 3XTgAD mice treated with HLJDT-M or HLJDT or vehicle (**c** and **d**). The statistical significance are denoted as *p < 0.05, **p < 0.01, **p < 0.001 when compared with vehicle-treated 3XTg-AD mice. Data represent mean ± SEM. N* = *8 mice per group.

**Figure 6:**
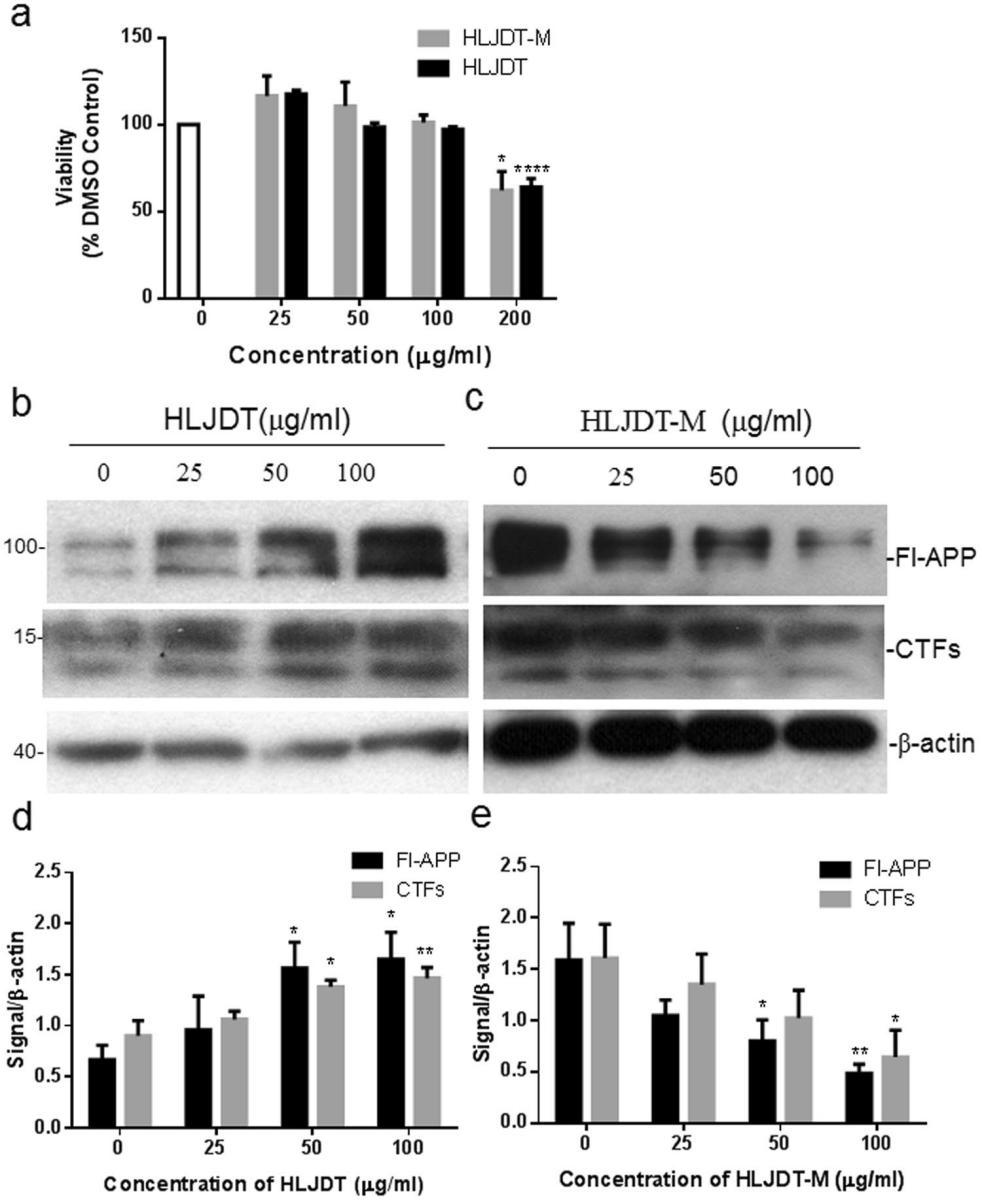
Treatment of N2a-SwedAPP cells with HLJDT-M and HLJDT differentially modulated the processing of APP. Cells incubated with aqueous extract of HLJDT or HLJDT-M at three different concentrations (25, 50 or 100 μg/ml) or with vehicle for 48 hours. After incubation, cell lysates were probed for APP and CTFs by Western blot. The statistical significance among different groups are represented as *p < 0.05, **p < 0.01, **p < 0.001.

To understand the different regulatory effects of HLJDT and HLJDT-M on memory function and Aβ plaque formation in 3XTg-AD mice, we examined the levels of APP, CTFs, and their phosphorylated forms. The phosphorylation of APP at threonine 668 (pAPP) has been proposed as a key event for APP maturation, subcellular distribution of APP-CTFs and generation of Aβ^18–20^. These studies have also shown that the APP intracellular cytoplasmic domain (AICD) with phospho-Thr668 accumulates in the brains of AD patients as well as in AD mice, and it mediates the interaction of Aβ and tau^21^. Therefore, we probed and estimated the levels of pAPP and pCTFs in SDS lysates by immunoblot analysis applying a polyclonal antibody against pAPP at Thr668. The SDS fraction was subjected to Western blotting to analyze the levels of FL-APP, pAPP, CTFs, pCTFs, pTau (PHF-1 epitope) and total human Tau (HT7). The levels of APP/CTFs, pAPP/pCTFs, PHF-1 Tau and total Tau were significantly elevated in 3XTg-AD mice when compared with the approximate WT (B6129SJ/F2) control mice (Supplementary Fig. 4). Densitometric analysis of the signal intensities of FL-APP and CTFs revealed decreases to the levels of 28% (p < 0.01) and 42% (p < 0.05), respectively, after HLJDT-M (2 g/kg) treatment (Fig. 5a,b). Quantitative analysis of pAPP and pCTFs showed 39–50% (p < 0.01) decrease in pAPP and pCTFs in the 1 g/kg HLJDT-M treatment group, and 43–44% decrease in pAPP and pCTFs in the 2 g/kg HLJDT-M group compared with the vehicle group (Fig. 5a,b). In contrast, 1 g/kg of HLJDT significantly augmented the levels of FL-APP, pAPP, CTFs, and pCTFs to a greater extent than 1 g/kg of HLJDT-M treatment (Fig. 5c,d). HLJDT treatment at a dose of 1 g/kg increased the levels of APP, pAPP, CTFs and pCTFs by 121% (p < 0.05), 120% (p < 0.001), 126% and 179% (p < 0.001), respectively (Fig. 6c,d). The maturation and subcellular localization of APP could be controlled through the phosphorylation of APP and CTFs^19^ because this phosphorylation regulates the generation of CTFs and Aβ^22^. It is interesting to note that HLJDT strongly increased and HLJDT-M significantly decreased the levels of pAPP and pCTFs, suggesting that 6 months of HLJDT-M consumption could have neuroprotective effects in APP mice via decreases in the levels of pAPP and pCTFs.

Considering the inhibitor role of HLJDT-M on FL-APP accumulation and its phosphorylation, we attempted to determine a possible role of HLJDT-M or HLJDT in Tau accumulation and its hyperphosphorylation in the 3XTg-AD mice. It has been reported that phosphorylated Tau at AT8 and PHF-1 epitopes were apparent in CA1 region between 15 and 18 months of age in 3XTg-AD mice^15^. In our mice colony, AT8 or PHF-1 positive neurons became evident only at 18 months of age (data not shown). In the present study, 3XTg-AD mice were 13 months of age at the end of experiments and we did not observe significant staining of hyperphosphorylated Tau, demonstrating that these mice were still to develop tau pathology. However, in Western blotting analysis, we found that only HLJDT-M treatment significantly decreased both the levels of PHF-1 Tau and human total Tau in 3XTg-AD mice brain lysates. We observed a significant reduction in the PHF-1 Tau in the brain lysates of HLJDT-M-treated 3XTg-AD mice (Fig. 5a). Densiometric analysis of PHF-1 and total Tau showed 42% (p < 0.01) and 32% (p < 0.05) reduction respectively, by 2 g/Kg/d of HLJDT-M relative to vehicle-treated 3XTg-AD mice (Fig. 5b). HLJDT treatment neither influenced the phosphorylation of Tau nor significantly affected total Tau (Fig. 5b,d). Since the study ended too early to see major effects on Tau pathology (PHF-1-immunoreactive dystrophic neurites or neurofibrillary tangles), it is recommended to explore the long-term effects of HLJDT-M on Tau pathology in aged 3xTg-AD mice.

### HLJDT and HLJDT-M alter APP processing by cells *in vitro*

Given the differential role of HLJDT and HLJDT-M in the modulation of APP processing in the 3X-TgAD mouse brain, we further evaluated APP processing in N2a-SwedAPP cells following HLJDT or HLJDT-M treatment. In our previous study, we showed that ethanol extracts of HLJDT and HLJDT-M have different effects on APP processing and Aβ generation^11^; here we confirm this effect using an aqueous extract of HLJDT and HLJDT-M. At first, we performed the MTT assay to measure the viability of N2a-SwedAPP against the HLJDT and HLJDT-M treatment for 48 h. The MTT crystal formation in cells was used to determine the viability of HLJDT and HLJDT-M treatments on N2a-SwedAPP cell lines. Compared with vehicle treatment, HLJDT and HLJDT-M showed no significant effects on the crystal formation till the concentration of 100 μg/ml (p > 0.05) (Fig. 1c), but the 200 μg /ml of extracts significantly decreased the formation of MTT crystals in the N2aSwedAPP cells conditioned medium (P < 0.001). Therefore, we tested the APP modulatory effects of HLJDT-M- or HLJDT up till 100 μg/ml.

Using CT695 antiserum, immunoblot analysis of cell lysates showed a dose-dependent rise in the levels of FL-APP and CTFs after HLJDT treatment (Fig. 6), whereas HLJDT-M treatment showed a decreasing effect on APP and CTFs. The maximal level of FL-APP increased by HLJDT was observed between 50 and 100 μg/mL (2- to 2.5- fold of basal release; p < 0.05). HLJDT increased CTFs to 1.5 and 1.65 times the basal levels at concentrations of 50 (p < 0.05) and 100 (p < 0.05) μg/mL, respectively. In contrast, HLJDT-M dose dependently and significantly reduced the level of FL-APP by 50% (p < 0.01) and 70% (p < 0.001) at concentrations of 50 and 100 μg/ml, respectively (Fig. 6b). HLJDT-M also decreased the level of CTFs by 37% and 60% (p < 0.05) at concentrations of 50 and 100 μg/ml, respectively (Fig. 6). The tested concentrations of HLJDT-M or HLJDT had no effect on the viability, multiplication rate or the cell morphology of cell lines (data not shown).

## Discussion

HLJDT-M could be a potential TCM recipe for treating AD because it does not have the β-amyloid increasing effect of HLJDT, as demonstrated in *in vitro* and *in vivo* studies^8^. So far, the concrete therapeutic value of HLJDT-M or HLJDT in terms of Aβ pathology and memory impairments has not been validated. For the first time, our study reveals that HLJDT-M treatment decreases Aβ plaque deposition, and recovers both spatial learning and memory retention impairments in 3XTg-AD mice. The patented herbal formulation of HLJDT-M is composed of RC, CP and FG at the ratio of 4:2:4, and it is responsible for significantly decreasing all APP metabolic products, including Aβ (Fig. 5). Both 1 g and 2 g/Kg doses of HLJDT-M greatly decreased the memory dysfunction of 3XTg-AD in the spatial learning reference memory task as well as in long-term (24 h) spatial location memory retention tasks (Fig. 2a,c,d). Long-term HLJDT-M treatment did not show significant adverse side effects on general motor ability, exploration behavior, visual acuity and motivation, as demonstrated by visible platform and open field experiments (Supplementary Fig. 1b).

Notably, HLJDT-M-mediated amelioration of memory deficits is reflected by a significant reduction in the plaque load. The 35% reduction in Aβ plaque load observed in 3XTg-AD mice treated with 1 g/Kg dose of HLJDT-M is also comparable to the level of BBR-mediated reduction of Aβ plaque load in TgCRND8 mice^10^. Differential extraction of brain tissues by SDS and FA is a generally used protocol to quantify the detergent-soluble and acid-soluble Aβ species, respectively, in different AD mouse models^17, 23^. In this study, we have observed that both 1 g and 2 g/Kg doses of HLJDT-M significantly reduced both detergent-soluble and acid-soluble Aβ species in the brain lysates of 3XTg-AD mice (Fig. 4). This reduction restores levels of insoluble Aβ species to levels comparable to those in the brain lysates of BBR-treated TgCRND8 mice^10^.

Notably, both 1 g and 2 g/Kg doses of HLJDT-M reduced both soluble Aβ1-42 (by 37–39%) and insoluble Aβ1-42 (32–66%); therefore, HLJDT-M has an Aβ-reducing effect on both detergent-soluble Aβ oligomers and -insoluble Aβ fibrils. Overall, the HLJDT-M-induced reduction of total extracted Aβ1-42 (37–66%) in the lysates from the one brain half hemisphere correlates well with the 35% decrease in Aβ plaque burden in other hemispheres as estimated by immunostaining. These results signify that these ELISA and immunohistochemical analyses reflect the total Aβ plaque load (Figs 3 and 4). Since a progressive shift of brain Aβs from soluble to insoluble pools plays a mechanistic role in the onset and/or progression of AD^24^, the ability of HLJDT-M to mediate a decrease in insoluble Aβ1-42 may explain its ability to delay the onset of AD.

We have found that HLJDT-M has a high amount of the protoberberine alkaloid BBR (Fig. 1). Previously we have shown that BBR can decrease Aβ by reducing the accumulation of phosphorylated APP and CTFs^10^. Since phosphorylated APP plays a key role in the maturation and subcellular trafficking of APP, CTFs and AICD, the pAPP-reducing effect of HLJDT-M substantiates its anti-AD effect.

Although HLJDT-M contains other protoberberine alkaloids such as palmatine, phellodendrine, epiberberine coptisine and jaterorhizine in a moderate amount, we ascertained that it is mainly BBR that accumulates in the brain tissues at a relatively high concentration (data not shown). This concentration is consistent with that of berberine in rat hippocampus, which was much higher than that in the hippocampus after intravenous administration of RC extract^25^. Therefore, HLJDT-M mediated reduction of both the plaque load and memory impairment is probably due to the presence of higher amounts of BBR in HLDT-M.

By contrast with HLJDT-M, HLJDT treatment increases APP and Aβ and does not protect memory in 3XTg-AD mice. Further, HLJDT did increase APP processing and Aβ plaque formation in 3XTg-AD mice. Both the soluble and insoluble levels of Aβs were raised in ELISA experiments. Recently we have shown that treatment with HLJDT, RS or their pure compound baicalein could increase APP metabolites, including Aβ, by modulating APP processing in N2a-SwedAPP cells. This study confirms that HLJDT treatment in an animal model of AD increases APP and Aβ. The HLJDT-mediated increase in the levels of APP and Aβ observed in this study is comparable with the baicalein-mediated increase in the levels of APP and Aβ in TgCRND8 mice^11^. In contrast to our studies, Qiu *et al*. have shown that oral administration of HLJDT (0.86 g/kg) slightly reduced the congo-red stained Aβ plaques and improved memory in APP/PS-1 mice^26^; however, they did not quantify the Aβ plaque load, and there was no statistical analysis of Morris water maze experimental data in their study. They also found that HLJDT reduced murine Fl-APP mRNA level without determining the effect of HLJDT on the levels of human APP, pAPP, CTFs and Aβ^26^. Our study using a well- known AD transgenic model mice and cell systems has conclusively found that HLJDT stimulates the amyloidogenic pathway, suggesting that this drug might not be effective for protecting neurons from neurodegeneration. Therefore, the outcome of our study provides clear evidence that HLJDT increases APP and Aβ.

Moreover, we have recently confirmed that RS is accountable for the APP/Aβ enhancing effect of HLJDT^11^, because the exclusion of RS from HLJDT restored its anti-APP/Aβ activity in the form of HLJDT-M and RS itself had a strong APP-increasing effect^11^. In this study, we found that baicalin and wogonoside are major glycosidic flavonoids in RS and HLJDT containing RS (Fig. 1a). Other studies have shown that dihydroflavone a metabolite of baicalin, is also found in RS^27, 28^. Baicalin and wogonoside are two major, naturally occurring flavone glycosides and they are natural prodrugs which are generally presumed to be absorbed as aglycones (baicalein and wogonin) in the large intestine after being hydrolysed by intestinal microflora through their β-glucuronidase activity^29^. In contrast, few studies have reported that some amount of aglycones is regenerated to the parental form (glycosides) in the liver through the conjugation of glucuronic acid with agylones^30, 31^. Although there are few reports on the neuroprotective effects of baicalin against Aβ-induced memory loss in rodent models^32, 33^, aglycone (baicalein) or a metabolite (dihydroflavone) of baicalin may hinder the activity of baicalin. For example, we have shown that baicalein, a pure compound of RS, increased the Aβ load by upregulating the levels of APP and CTFs in both cellular and animal models of AD^11^ and during the same study we also found that wogonin also increased the levels of Fl-APP (data not shown). In line with our findings, Zhou *et al*. have shown that the chronic treatment with 7,8 dihydroxyflavone, a metabolite of baicalin, not only slightly increased the numbers of Aβ plaque load but also slightly upregulated the levels of FL-APP/CTFs without affecting cognitive impairment of APP23 transgenic mice^34^. Moreover, they have also found that dihydroxyflavone slightly augmented the levels of Fl-APP and CTFs in both HEK293-Swedish APP and SHSY5Y cell lines^34^. Therefore, we propose that aglycone or dihydroflavone metabolites of baicalin may inhibit the degradation of APP/CTFs that leads to increased production of Aβ in transgenic mice models of AD. A detailed mechanistic study is needed to reveal how the aglycones or metabolites of baicalin are responsible for the increase of Aβ/Fl-APP.

Since HLJDT is currently being administered as an adjuvant medicine to control the aggressiveness of aged AD patients^35^, our discovery that HLJDT enhances Aβ production evokes the concern of likely side-effects and of hastening AD clinical manifestations, especially in the aged. While the particular mode of action of HLJDT in the AD remains mostly unidentified, recent studies^36, 37^, together with ours^11^, propose that baicalein from HLJDT crosses the blood brain barrier and induces Aβ-increasing effects upon chronic administration.

In conclusion, our data indicate that the likely APP-increasing effects of HLJDT on AD patients perhaps avoided by removing RS from it (i.e., HLJDT-M) and by using an appropriate ratio of RC, CP and FG. This new formulation could be beneficial in mitigating AD progression. Since we did not test the appropriate weight ratio of RC, CP and FG ratio in HLJDT, an unbiased mathematical space-filling design named “Uniform Design”^38^ should be applied to tabulate the different ratios by weight of the three herbs, and evaluate the anti-AD activity of the different formulations of HLJDT-M *in vitro*. Then the combinations of herbal components of HLJDT-M should be assessed by direct testing of the formulation in animal models. This traditional form of medicine, based on a new effective formula, manufactured with stringent quality control, should yield a safe, effective new pharmaceutical for AD—a disease for which, so far, no drug has been developed. Although HLJDT has been prescribed by TCM practitioners to patients with cerebrovascular disease, a multicenter, double-blind, clinical study of HLJDT-M should be done to evaluate the complete efficacy of HLJDT-M against AD. Moreover, whether HLJDT-M ought to be used alone or co-administered with FDA-approved western medicine should be evaluated in randomised clinical trials.

While there is much to be done, the evidence from our study suggests that this new, revised formula of a time-honored medicine, if manufactured with stringent quality control, should yield a safe, effective new pharmaceutical for AD—a disease for which, so far, no drug has been developed. According to the amyloid hypothesis of AD etiology, the excessive generation and aggregation of Aβ are primary pathogenic events in AD. Although this hypothesis has prompted an extensive search for therapeutic agents to modulate the generation of Aβ, all clinical trials with these objectives have so far failed. A possible reason for these failures is that, by the time AD dementia is evident, the progression of neuropathology may have reached an irreversible stage. To test this idea and attempt to overcome this obstacle, trials are ongoing in people who are genetically susceptible to AD but who have not yet become demented. If those trials are successful, our HLJDT-M formulation could have a big advantage over the drugs in those trials in terms of cost effectiveness and safety. Since HLJDT has already been used in clinics for over 1700 years in China and Japan for cerebrovascular disease, it should be relatively straightforward to translate the modified HLJDT into a clinical drug. Further, this study will lay a foundation for the modernization of Chinese medicine and for the development of an anti-AD cocktail based on TCM theory.

## Methods

### Reagents and antibodies

Monoclonal antibody 6E10 (BioLegend, San Diego, CA, USA), which recognizes amino acid residues 1–16 at the N-terminus of APP, and which detects the Aβ was purchased. Monoclonal antibody 4G8 (Biolegend), which recognizes amino acid residues 17–24 at the N-terminus of APP and which detects the Aβ, was purchased. Biotinylated mouse monoclonal antibodies to human Aβ1-40 (5C3) and human Aβ1-42 (8G7) were purchased from Nano tools (Teningen, Germany). Polyclonal antibody pAPPT668 (Cell signaling, Danvers, MA, USA), which recognizes the phosphorylated APP at Threonine 668 residues, was purchased. Polyclonal anti-APP antibody (CT-696), which recognizes the C-terminus of APP, was purchased (Thermo Scientific, Waltham, MA, USA). β-actin antibody was acquired from Cell Signaling (Danvers, MA USA). Synthetic Aβ1-40 and Aβ1-42 peptides were acquired from Thermo Scientific and American Peptide (Thermo Scientific), respectively. The Streptavidin-HRP conjugate was obtained from Dako (Agilent, Santa Clara, CA, USA).

### Preparation and quality analysis of HLJDT and HLJDT-M

The drug materials of RC, CP, RS and FG were procured from the Mr. & Mrs. Chan Hon Yin Chinese Medicine Specialty Clinic and Good Clinical Practice Centre (affiliated to Hong Kong Baptist University [HKBU]) and identified as stipulated by the Chinese Pharmacopeia (2010 Edition). The dried herb specimens were deposited at the School of Chinese Medicine, HKBU, Hong Kong, China. HLJDT was prepared by mixing RC, RS, CP and FG in the ratio of 3:2:2:3. HLJDT-M was prepared by mixing RC, CP and FG in the ratio of 4:2:4. The total weight of each formulation was 1 kg. Each formulation was decocted thrice by refluxing with water (1:10 w/v) for 1 h, and the solution obtained was filtered and concentrated using a rotary evaporator at 60 °C. The concentrated extract was frozen at −80 °C and lyophilized at −50 °C. The resultant yield of each formulation was pulverized and then stored at −20 °C until used.

Identification of the chemical constituents of each batch of HLJDT and HLJDT-M was performed using HPLC-TOF/MS as described earlier by us with little modification^11^. In detail, an aliquot of HLJDT and HLJDT-M was dissolved in methanol and centrifuged at 10,000 rpm; the supernatant was filtered and used for analysis. An Agilent 1290 UHPLC system (Agilent Technologies) was applied for chromatographic analysis. The soluble extracts were fractionated by an ACQUITY UPLC BEH C18 column (2.1 mm × 100 nm, 1.7 μm) at 40 °C. The mobile phase consisted of 0.1% formic acid in water (A) and 0.1% formic acid in ACN (B). The separation was conducted with a programmed gradient elution as follows: 0–10 min, 5% B; 11–13 min, 5–36% B; 14–21 min, 36–50% B; 21–22 min, 50–75% B; 22–22 min, 75–100% B; 23–27 min, 100% B; 27–27 min, 100% B; 27–30 min. The flow rate was 0.4 mL/min. The column temperature was 40 °C. An Agilent 6540 Q-TOF mass spectrometer (Agilent Technologies) connected with an electrospray (ESI) ion source was applied to obtain MS and MS/MS data in positive ion mode. Data acquisition and data integration was attained by MassHunter B.03 software (Agilent Technologies). Mass spectra were documented for a mass range of 100–1700 m/z for all mass peaks. The injection volume was 2 μl for MS analyses.

### Animals study

Animal experiments such as breeding, colony maintenance and drug administration were perfomed in the animal house of Hong Kong Baptist University, and the procedures were approved by the Human and Animal Subjects in Teaching and Research (HASC approval # HASC/13–14/0165), Hong Kong Baptist University. The behavior experiments were performed in the AAALC-certified laboratory animal unit of the University of Hong Kong under protocols approved by the Committee on the Use of Live Animals for Teaching and Research (CULATR #3314). All animal experiments were performed in accordance with the relevant guidelines and regulations of both HASC and CULATR. Triple transgenic mice (3XTg-AD), carrying three mutant transgenes, i.e., amyloid precursor protein (Swedish, K670M/N671L), presenilin-1 (M146V), and tau (P301L), were used as an AD mouse model^10^. 3XTg-AD mice, C57BL6J and 129svJ were purchased from the Jackson Laboratory (Bar Harbor, ME, USA). The approximate WT type control B6129F2 was generated by crossing C57BL6J and 12svJ mice and the resulting F1 generation, B6129F1 were intercrossed to the get the B6129F2. The animals were housed in a pathogen-free facility under 12 hour light, 12 hour dark cycles with food and water provided ad libitum. Seven-month old 3xTg-AD mice were separated into five different groups (n = 16 mice per group with equal sex). Oral administration of HLJDT and HLJDT-M started at 7 months of age and completed at 13 months of age. Both formulations were orally administered to 3XTg-AD mice by gavage every other day at two different doses of herbal extracts, that is 1 g/kg/d and 2 g/kg/d for 6 months until the conclusion of the study. The tap water was given to the vehicle control group. The doses of HLJDT were selected based on the previous studies^11^. The body weight, coat color and texture, and in-house behavior were observed during the study. After 6 months of treatment, the number of males and females mice in each group is as follows: (i) Tg-vehicle group (8 males and 7 females); (ii) Tg-HLJDT-M 1 g/Kg group (7 males and 7 females); (iii) Tg-HLJDT-M 2 g/Kg group (5 males and 7 females); (iv) Tg-HLJDT 1 g/Kg group (6 males and 7 females); and Tg-HLJDT-M 2 g/Kg group (7 males and 6 females). A sixth group of 7-month old WT mice (n = 12 mice per group with equal sex) was included as a Non-Tg vehicle group and there is no change in the number of animals in this group after the treatment.

### Open field test

The open field test was done as detailed in Zhang *et al*.^39^. The apparatus was a square plexiglass box (25 × 25 cm). The marginal area was selected as being within 10 cm from the walls. Each mouse was positioned at a corner and allowed to move freely, and the behavioral of activity of each mouse were recorded for 5 min. Mouse behavior parameters such as walking distance, velocity, time spent in movement and duration spent in central/marginal areas were recorded and analyzed by the Ethovision tracking system.

### Morris water maze test

The Morris water maze (MWM) test was conducted as detailed by us and others^10, 13^. The MWM consists of a circular, 1 m diameter pool made of non-toxic white plastic, filled with water and maintained at 21 ± 1 °C. Animals were housed in the behavior room, acclimatized for 10 days before starting the experiments. Swim paths were monitored using an automated tracking system (EthoVision video tracking system (Version 3.0, Noldus Information Technology, Leesburg, VA, USA). Before all learning and memory tests, visible platform trials were performed to test whether drug administration could cause alternation in visual acuity that might influence performance during the learning task. For visible platform training, the platform was placed above the water level with a flag attached, and the platform position was changed for each trial (4 trials per day). Hidden platform training consisted of 6 sessions (1 per day) over 6 days; each session comprised four 60 second trials with a 30 min inter-trial interval. The platform location remained unchanged in the hidden-platform trials, and the entry point was changed randomly between days. A probe trial was performed at 24 h after the 6^th^ hidden-platform training trial to assess long-term memory retention. The probe trial was conducted without the platform, and mice were allowed to search the platform for 60 seconds. The amount of time spent in the target quadrant (quadrant where the platform was originally placed) in comparison with the time spent in the remaining three quadrants was taken as an indication of the level of long-term memory retention of the 3XTg-AD mice. The distance moved (path length) to reach the platform and the total time spent in each quadrant of the pool in the probe trial by each mouse were recorded and analyzed using EthoVision video tracking system.

### Immunohistochemistry

Immunohistochemical staining, image analysis of Aβ plaques and quantification of Aβ load were conducted as detailed by us ref. 10. The mice were sacrificed by CO_2_ anaphylaxis. After brains were carefully removed, the right hemisphere portion was snap-frozen for biochemical study, while the left hemisphere was fixed in 4% paraformaldehyde at 4 °C for 2 days. The fixed brain tissues were rinsed twice with 1X PBS, cryoprotected in 30% sucrose at 4 °C for 2 days, and finally embedded in OCT (optimal cutting temperature) compound. Coronal, frozen, 30 μm sections from the anterior, medial, and posterior regions of the cortico-hippocampal region were cut on a cryostat at 120-μm intervals and stored at 4 °C in PBS with 0.01% sodium azide. Three sections from each region were immunostained using a mouse anti-human amyloid-β monoclonal antibody conjugated to biotin (4G8; 1:1000; BioLegend).

### Measurements of Aβ species by ELISA

To detect overall level of Aβ species among treatment groups, the right hemispheres of the brains of the mice were subjected to sequential extraction first with SDS and then with FA for determining the detergent-soluble Aβ and formic acid-soluble Aβ, respectively, as previously described^10, 23, 39^. Briefly, the brain tissues were first homogenized in 10 volume of 2% SDS in TBS with appropriate amount of protease inhibitor cocktail and Phostop cocktail tablets (Roche, Basel, Switzerland). The resulting SDS lysate was centrifuged at 100,000 × g for 1 h at 4 °C to obtain a detergent-soluble fraction. The resulting pellets (detergent-insoluble fractions) were homogenized in 70% FA in 50 mM TBS PH 7.4, followed by a short sonication (10 s). The resulting suspension was centrifuged (20,000 × g; 4 °C; 20 min), and 200 μL of the supernatant was neutralized with 1:12 dilution of Tris-base 2 M (pH 11) to be used for ELISA. The levels of SDS- and FA-soluble Aβ1-40 and 1–42 were measured by a sandwich ELISA as detailed by Durairajan *et al*.^10, 40^. A standard curve of Aβ1-40 and Aβ1-42 was constructed using synthetic pure peptides, and the unknown amount of Aβ species was measured in lysates using the standard curves.

### Cell culture, viability and APP modulation assay

N2a cells constantly expressing the human Swedish mutant APP (N2a-SwedAPP) were gifted by Dr. Gopal Thinakaran (University of Chicago, Chicago, IL, USA). N2a-SwedAPP cells were cultured in 1:1 DMEM/Opti-MEM supplemented with 5% FBS, 1X PSN and 200 μg/mL of genicitin/G418 (Thermo Scientific)^41^. For cellular viability assay, N2a-SwedAPP cells were plated at density of 10000 cells/well, on 48-well plates. After 24 h of incubation, the spent media was replaced with new media containing HLJDT or HLJDT-M at the final concentration indicated. Forty-eight hours after replacement of the drug media, 100 μl of phenol red-free DMEM containing MTT (Thermo Scientific) (5 mg/ml) was added to each well, and plates were incubated for a further 4 h. The MTT solution was then purged, and the cell crystals were dissolved using 100 μl of 20% SDS in 50% DMF mixture. Finally, color intensity was measured using an ELISA reader at 570 nm. The baseline was determined in control wells containing no cells and the acquired values were subtracted. The APP processing assay was performed as described by us previously^41, 42^. Briefly, N2a-SwedAPP cells were plated at densities of 2 × 10^4^ cells/well on 12-well plates. After 24 hours of culture, the spent media was replaced by new media containing HLJDT or HLJDT-M at the indicated final concentrations or DMSO control, and plates were incubated for a further 48 hours. The cells were lysed in ice-cold RIPA buffer (Cell Signaling) with protease inhibitors and 20 μg of cell lysates were subjected to 10 and 15% SDS-PAGE. The levels of FL-APP, CTFs, pAPP and β-actin in the cell lysates were quantified by Western blotting.

### Western blotting

To investigate the effect of HLJDT and HLJDT-M on the APP processing, levels of FL-APP, CTFs, pAPP, PHF-1 Tau, total human Tau, β-actin, pCTFs, and BACE1 were measured in the SDS fractions of the brain lysate by Western blotting. Table 1 lists the specifications of antibodies against APP, Aβ, BACE, phospho-Tau and total Tau used in this study. Protein concentrations of brain and cell lysates were assayed using BCA reagent (Thermo Scientific). Protein extracts from the brain (10 μg protein per sample) and the cell lysates (20 μg protein per sample) were resolved by 10%/15% SDS polyacrylamide gels. The proteins from the gels were electrophoretically transferred onto polyvinylidene fluoride (PVDF) membranes (GE Healthcare, Piscataway, NJ, USA). After transfer, the membranes were blocked with 5% skim milk fir 2 h at room temperature (RT) and then probed with the indicated primary antibodies at 4 °C for 18 h. After washing 3 times, the membranes were probed with horseradish peroxidase (HRP) conjugated affinity-purified secondary antibody: goat anti-mouse IgG, and goat anti-rabbit IgG (Jackson Immunolaboratories, West Grove, PA, USA). Protein signals were visualized using an ECL chemiluminescent HRP substrate detection system (Thermoscientific). Image analysis of the APP, pAPP, β-actin, PHF-1 Tau, total Tau, CTFs and pCTFs bands was performed with NIH Image J software.

**Table 1:** Specifications of antibodies used in this study.

Antibody (clone)	Region specificity	Antigen	Source	Use and Dilution
Rabbit polyclonal to APP CT695 (CT695)	Human, mouse and rat FL-APP and CTFs	C-terminus 22 amino acid residues of β-APP peptide	Thermoscientifi, Waltham, MA, USA	Western blotting (WB) 1:1000;
Mouse monoclonal to human Aβ1-16 (6E10)	human Aβ (hAβ)	Amino acids residues 1–17 of hAβ peptide	Biolegend, Dedham, MA, USA	ELISA capture: 4 μg/ml
Biotinylated mouse monoclonal to human Aβ17-24 (4G8)	hAβ	Amino acids residues 17–24 of hAβ peptide	Biolegend, Dedham, MA, USA	IHC 1:500;
Biotinylated mouse monoclonal to human Aβ1-40 (5C3)	C-terminus of hAβ1-40; does not crossreact with hAβ1-42	C-terminal hAβ1-40 peptide	Nano tools, Teningen, Germany	ELISA detection: 0.5 μg/ml
Biotinylated mouse monoclonal to human Aβ1-42 (8G7)	C-terminus of hAβ1-42; does not crossreact with hAβ1-40	C-terminal hAβ1-42 peptide	Nano tools, Teningen, Germany	ELISA detection: 0.5 μg/ml
Rabbit polyclonal to phosphorylated APP (Thr668)	Human Phosphorylated APP at Thr668	Phosphopepitopes matching to residues neighboring Thr668 of human APP695	Cell signaling, Danvers, MA, USA	WB 1:1000
Rabbit polyclonal to human BACE1 (ab2077)	Human and mouse BACE1	Amino acids residues 485–501 of Human BACE	Abcam, Cambridge, MA, USA	WB 1:1000
PHF-1 monoclonal to phospho Tau	Human, mouse and rat phospho Tau	Epitopes matching to residues neighboring Ser396 and Ser404 phosphorylated sites	Prof. Peter Davies Albert Einstein College of Medicine, Manhasset, NY, USA	WB 1:1000
HT7 monoclonal to total Tau	Human specific	Human Tau between residue 159 and 163	Thermoscientific	WB, 1:1000
Mouse monoclonal to β-actin (C4)	β-actin	Bird gizzard actin	Santa Cruz, Dallas, TX, USA	WB: 1:1000

### Statistical analysis

All data are represented as mean ± standard error of the mean (SEM). Analyses of behavioral data were performed using repeated-measures (“treatment” and “day”) 2-way analysis of variance (ANOVA). Data from the probe trial and immunhistochemistry was analyzed by One-way ANOVA analysis. Pair-wise differences between groups were compared using either Bonferroni’s or Fisher’s least significant difference (FLSD)-post hoc multiple comparisons test. All graphical presentation and statistical tests were executed with GraphPad Prism 6 (GraphPad Software, San Diego, CA, USA).

## Electronic supplementary material

Supplemenatary Information
