# Supplementary material for: A modified formulation of Huanglian-Jie-Du-Tang reduces memory impairments and β-amyloid plaques in a triple transgenic mouse model of Alzheimer’s disease
Source: Sci Rep. 2017 Jul 24;7:6238. doi: 10.1038/s41598-017-06217-9 (PMC5524904; doi:10.1038/s41598-017-06217-9)
Supplement: Supplementary file 1 — Supplemenatary Information [file 41598_2017_6217_MOESM1_ESM.pdf]

Supplementary Information

A modified formulation of Huanglian-Jie-Du-Tang reduces memory impairments and β-amyloid plaques in a triple transgenic mouse model of Alzheimer's disease

Siva Sundara Kumar Durairajan[1,2*¶], Ashok Iyaswamy[1,2¶], Sravan Gopalakrishnan Shetty[1,2], Ananth Kumar Kammella[1,2], Sandeep Malampati[1,2], Wenbin Shang[1,2], Chuanbin Yang[1,2], Juxian Song[1,2], Sookja Chung[3], Jiandong Huang[4], Kaliappan Ilango[5], Quan-Bin Han[6] and Min Li[1,2*]

Table 1. The analytical condition of LC-MS for the identification of the 12 compounds detected in HLJDT by LC-MS

Peak No.	Rt (min)	MS, m/z	Molecular formula	Identification
1	3.71	411.1262 $[M+Na]^+$	$C_{20}H_{24}NO_4$	Phellodendrine
2	3.99	342.1705 $[M]^+$	$C_{17}H_{24}O_{10}$	Geniposide
3.	4.11	227.01$[M]^+$	$C_{11}H_{14}O_5$	Genipin
4.	6.93	336.1230$[M]^+$	$C_{20}H_{18}NO_4$	Epiberberine
5.	7.03	338.1392$[M]^+$	$C_{20}H_{20}NO_4$	Jatrorrhizine
6.	7.12	320.0917$[M]^+$	$C_{19}H_{14}NO_4$	Coptisine
7.	7.86	447.0922$[M+H]^+$	$C_{21}H_{18}O_{11}$	Baicalin
8.	8.10	352.1543$[M]^+$	$C_{21}H_{22}NO_4$	Palmatine
9	8.11	336.1230$[M]^+$	$C_{20}H_{18}NO_4$	Berberine
10	9.25	461.1078$[M+H]^+$	$C_{22}H_{20}O_{11}$	Wogonoside
11	10.51	271.0601$[M+H]^+$	$C_{15}H_{10}O_5$	Baicalein
12	12.35	285.0757$[M+H]^+$	$C_{16}H_{12}O_5$	Wogonin

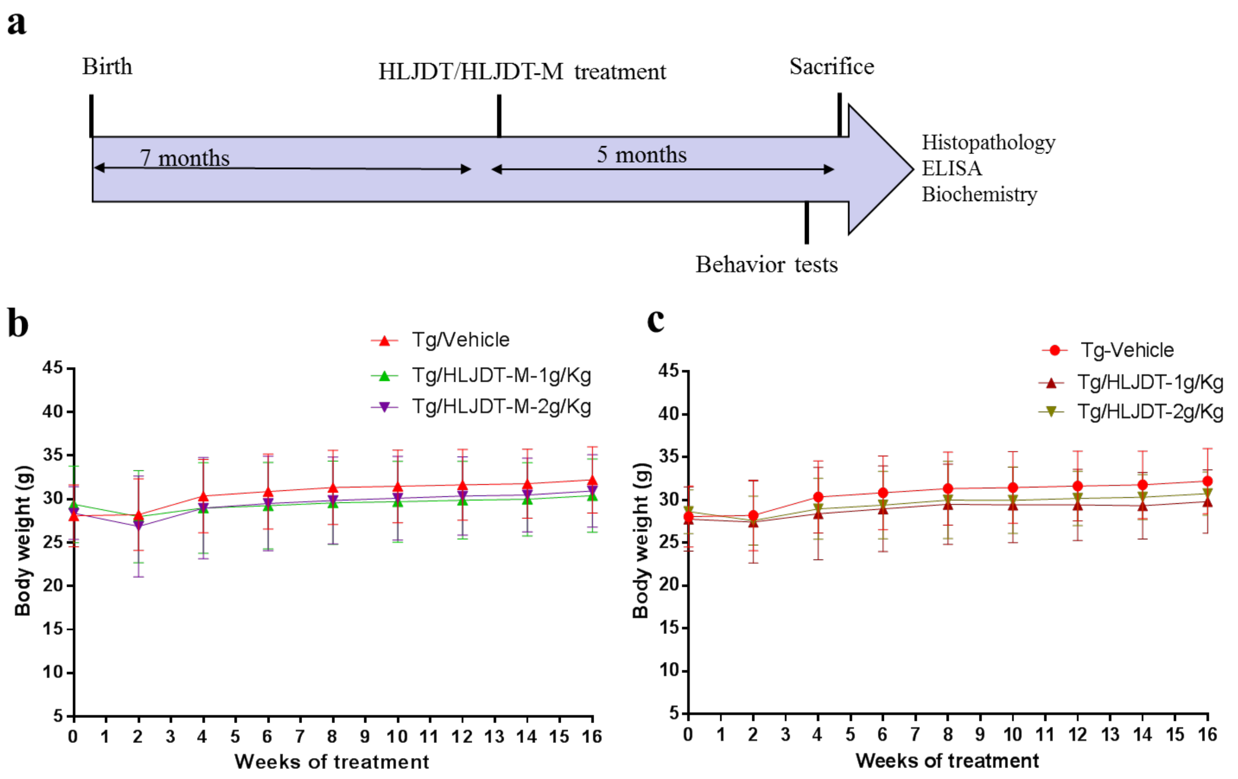

Figure 1. Chronic HLJDT-M and HLJDT treatment is highly tolerable and did not influence body weight in 3XTg-AD mice. HLJDT and HLJDT-M was orally administered from 6 to 13 months of age at 1 g/kg/d or 2 g/kg/d. The weight record is taken every two weeks for the first 4 months of treatment.

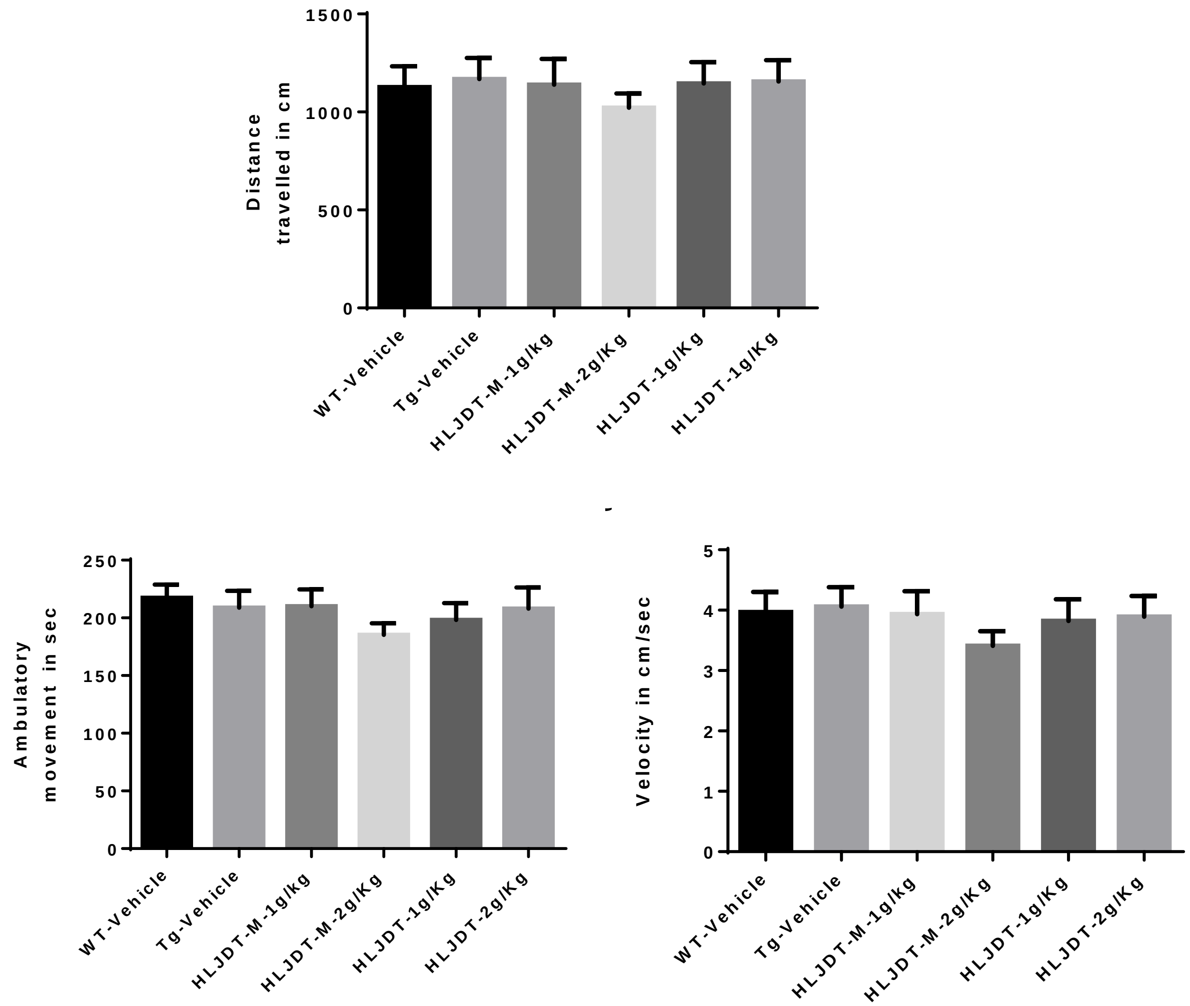

Figure 2. Chronic HLJDT-M or HLJDT treatment did not influence the overall locomotor activity in open field test. The WT, 3XTg-AD and drug treated mice showed similar total distance travelled (a), movement (b) and speed (c).

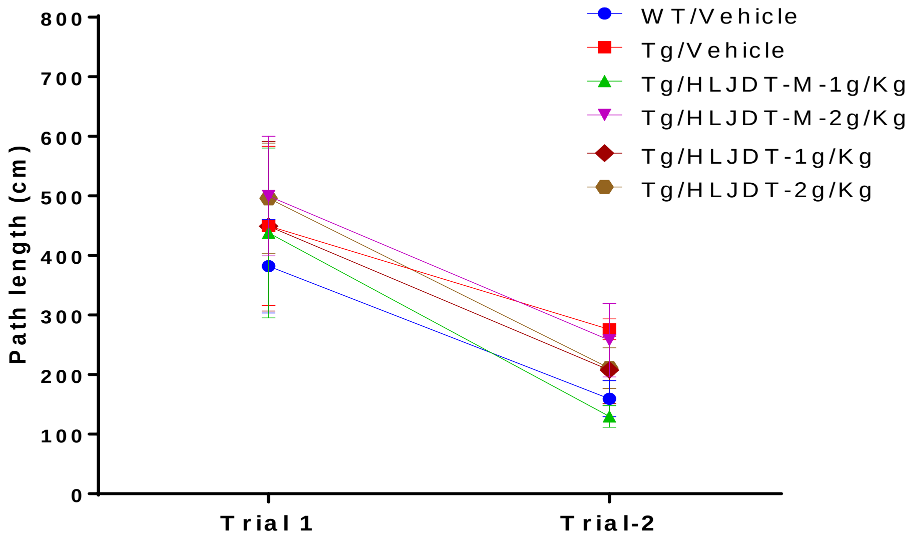

Figure 3. Chronic HLJDT-M or HLJDT treatment did not influence the visible platform learning trend in the Morris water maze.

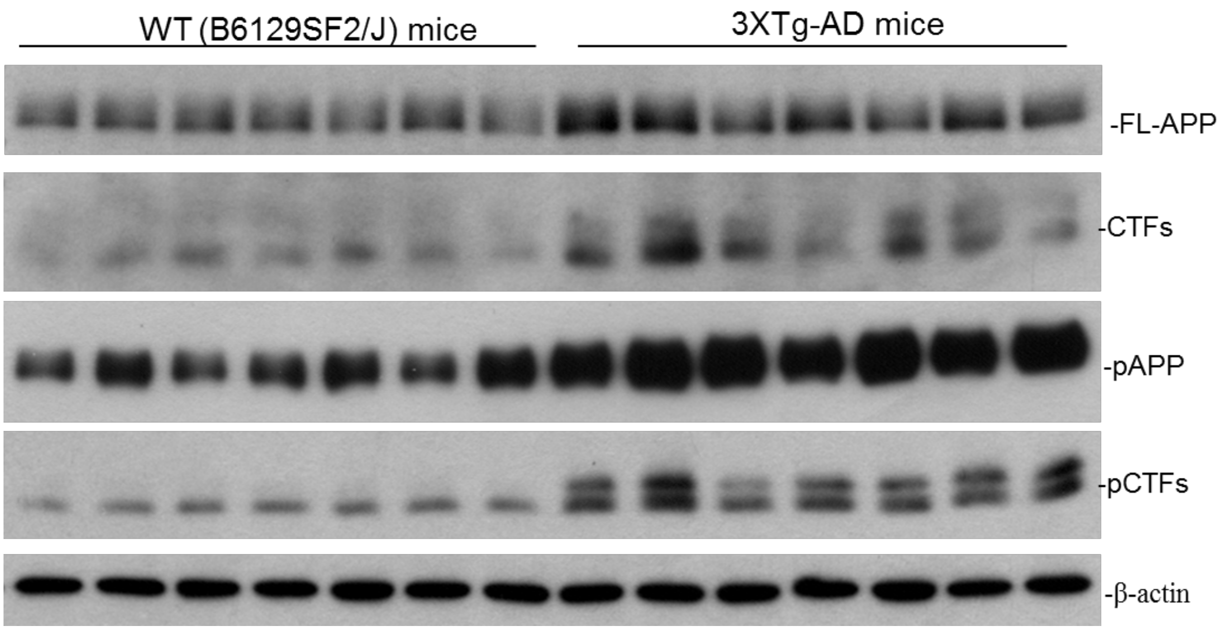

Figure 4. The level of APP, pAPP, CTFs, pCTFs in the brian lysates of WT (B6129SF2) and 3XTg-AD mice. Number of mice per group is 7.

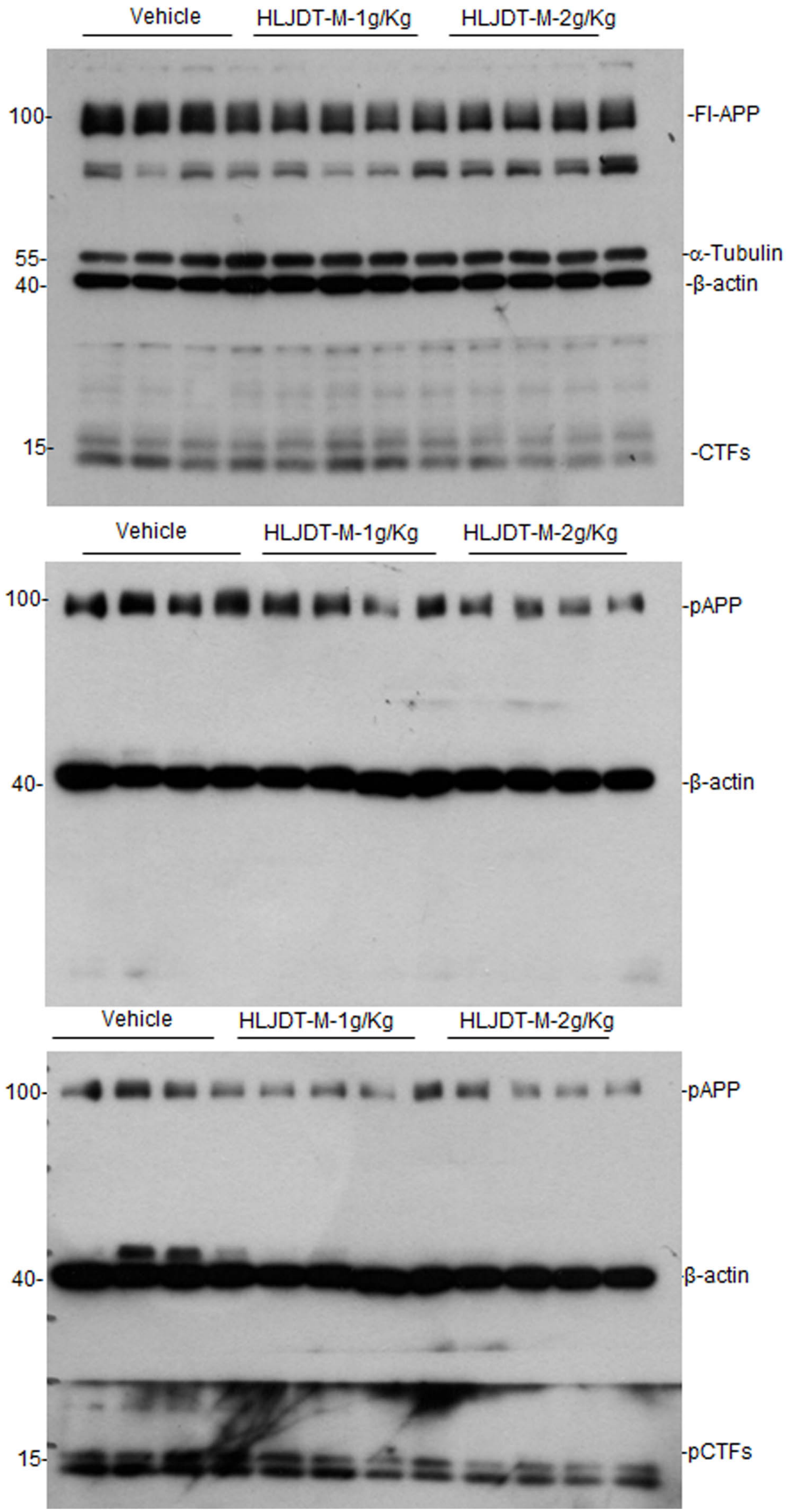

Figure 5. Full immunoblots of FL-APP, pAPP, CTFs, pCTFs and β–actin corresponding to the figure 5a.

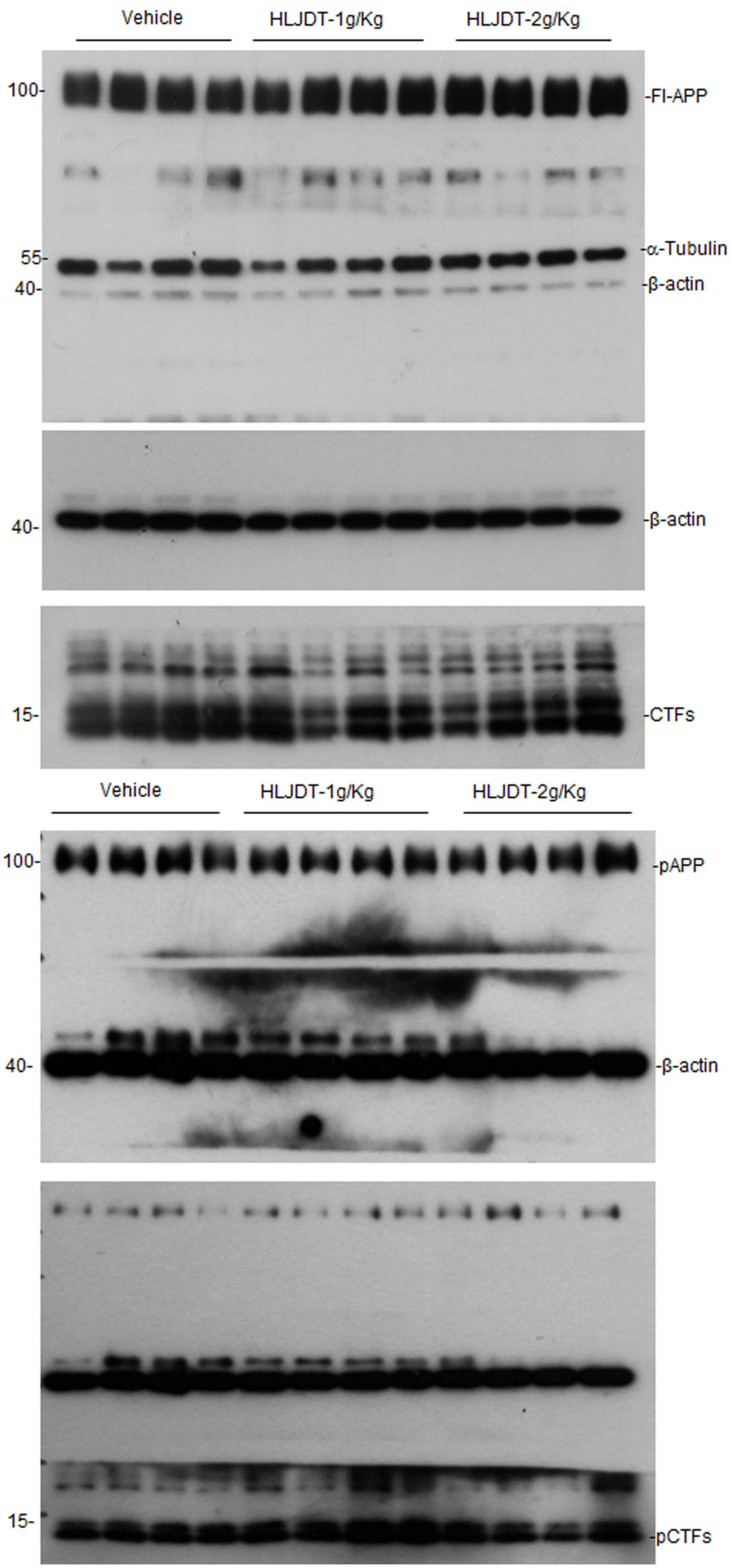

Figure 6. Full immunoblots of FL-APP, pAPP, CTFs, pCTFs and β–actin corresponding to the figure 5b.

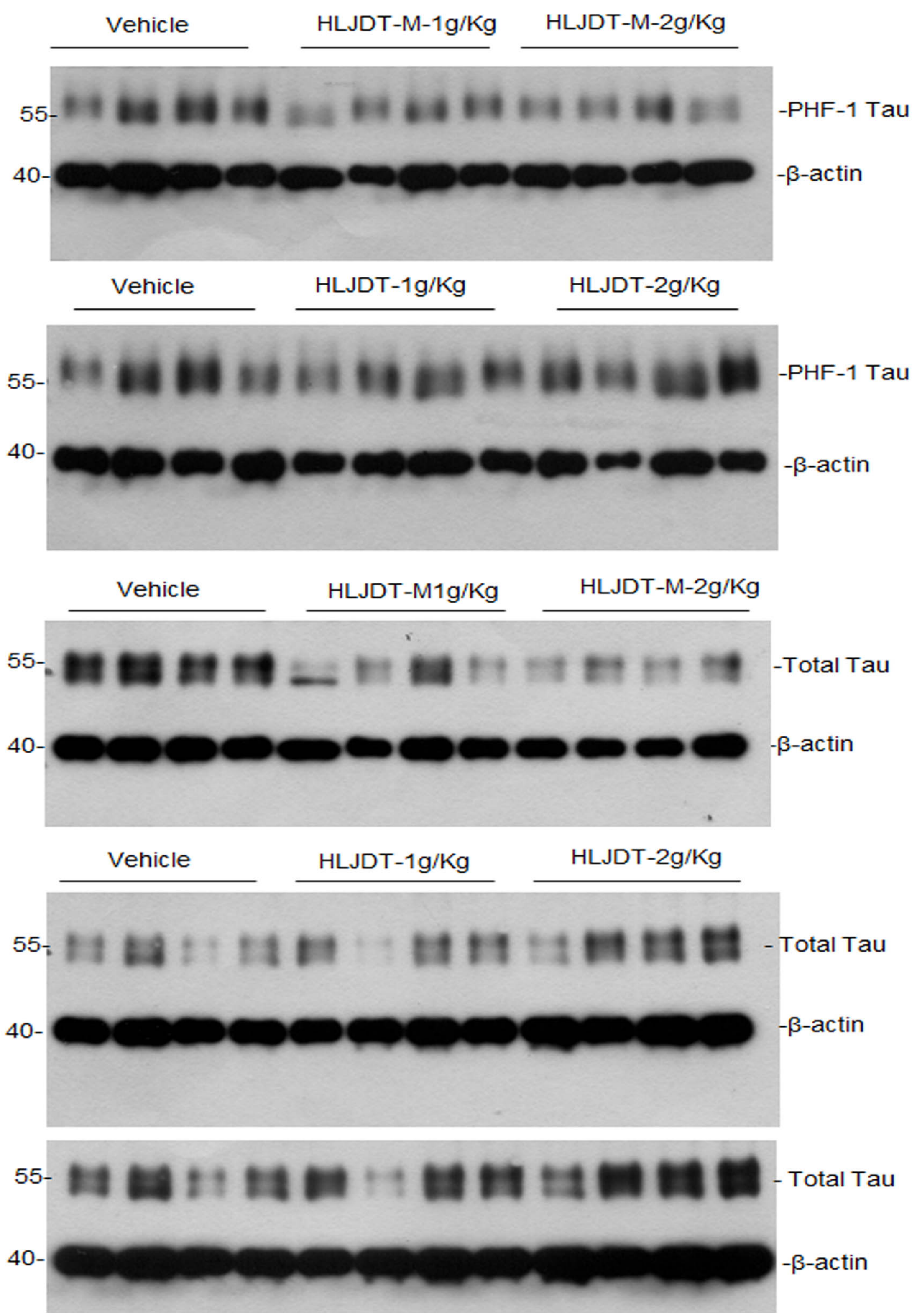

Figure 7. Full immunoblots of phospho-Tau (PHF-1) and total human Tau (HT7) and β–actin corresponding to the figure 5a and 5b.

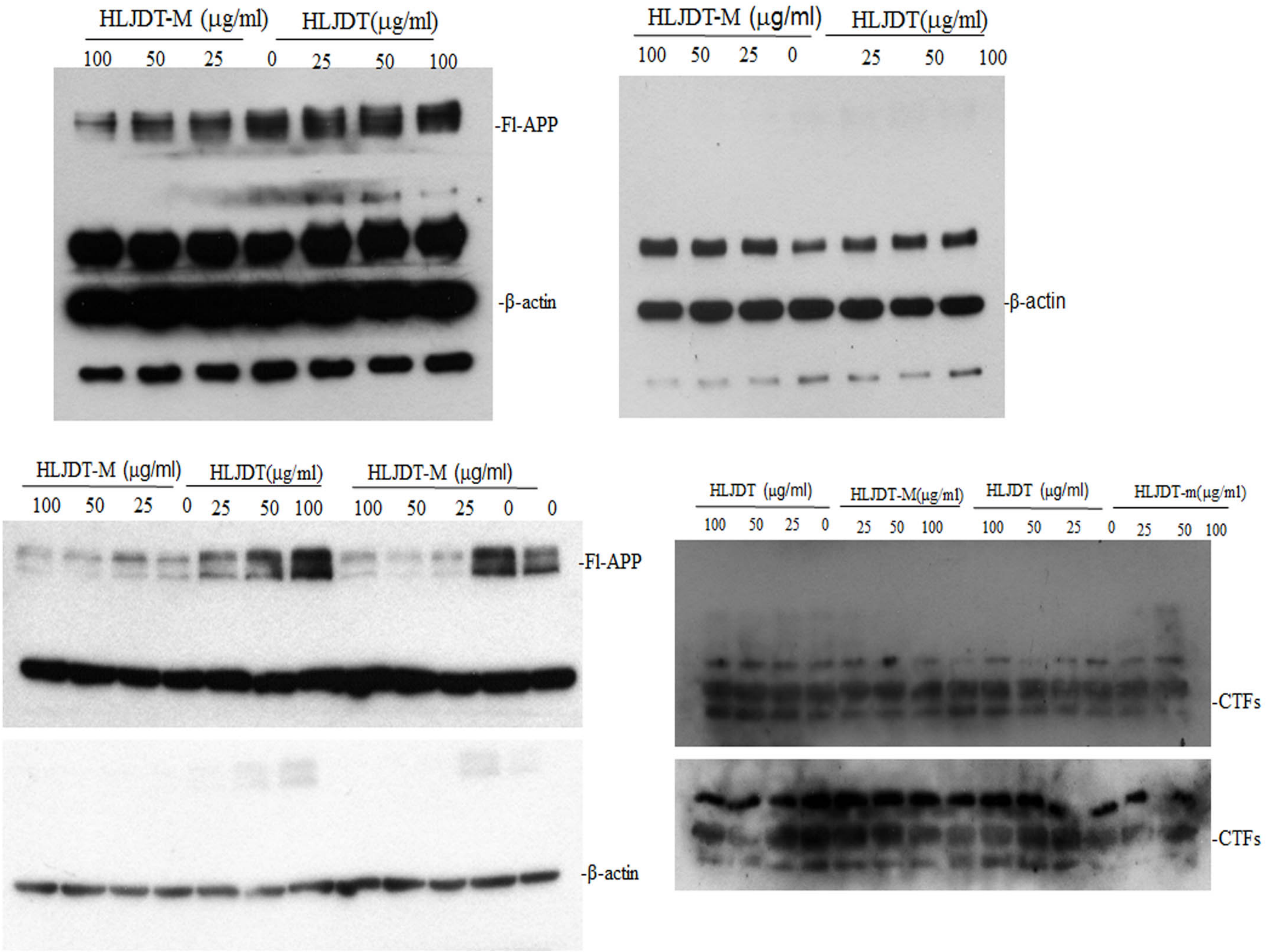

Figure 8.Full immunoblots of FL-APP, CTFs, and β–actin corresponding to the figure 6b and 6c.
